# Harvard Personal Genome Project: lessons from participatory public research

**DOI:** 10.1186/gm527

**Published:** 2014-02-28

**Authors:** Madeleine P Ball, Jason R Bobe, Michael F Chou, Tom Clegg, Preston W Estep, Jeantine E Lunshof, Ward Vandewege, Alexander Wait Zaranek, George M Church

**Affiliations:** 1 Department of Genetics, Harvard Medical School, 77 Avenue Louis Pasteur, Boston, MA 02215, USA; 2 PersonalGenomes.org, 423 Brookline Avenue, #323, Boston, MA 02215-5410, USA; 3 Curoverse, Inc., 51 Melcher Street, Boston, MA 02210, USA; 4 TeloMe Inc., 1393 Main Street, Waltham, MA, USA; 5 Section Molecular Cell Physiology, VU University Amsterdam, Amsterdam, The Netherlands

## Abstract

**Background:**

Since its initiation in 2005, the Harvard Personal Genome Project has enrolled thousands of volunteers interested in publicly sharing their genome, health and trait data. Because these data are highly identifiable, we use an ‘open consent’ framework that purposefully excludes promises about privacy and requires participants to demonstrate comprehension prior to enrollment.

**Discussion:**

Our model of non-anonymous, public genomes has led us to a highly participatory model of researcher-participant communication and interaction. The participants, who are highly committed volunteers, self-pursue and donate research-relevant datasets, and are actively engaged in conversations with both our staff and other Personal Genome Project participants. We have quantitatively assessed these communications and donations, and report our experiences with returning research-grade whole genome data to participants. We also observe some of the community growth and discussion that has occurred related to our project.

**Summary:**

We find that public non-anonymous data is valuable and leads to a participatory research model, which we encourage others to consider. The implementation of this model is greatly facilitated by web-based tools and methods and participant education. Project results are long-term proactive participant involvement and the growth of a community that benefits both researchers and participants.

## Background

The Personal Genome Project (PGP) was founded on the premise that highly integrated and comprehensive personal health information - in combination with personal genome data - is needed to understand the diverse functional consequences of genetic variation. George Church’s original proposal anticipated that such highly identifiable data collection efforts would raise issues with data sharing and security [1]. He suggested an alternative approach: avoid promising privacy, and recruit volunteers who understand the risks and want to make their personal data available to the public.

Much has happened in the years since the PGP was initiated nearly a decade ago. The cost of DNA sequencing has dropped another 10,000-fold - an individual genome is now as cheap as a personal computer. DNA sequencing has greatly expanded, not only resulting in improved interpretation of ancestry and trait information, but also in an ever-increasing potential for breaches of data security as more people have access to this data. A better understanding of the inherent identifiability of genetic data has also crystallized: individuals can be detected in aggregate samples [2], genotypes can be predicted from expression data [3], and genealogy databases can be used to infer surnames from Y-chromosome genetic data and re-identify dozens of ‘anonymous’ genomes [4]. There will always be some project-specific information that can be hard to obfuscate, for example, pedigree information based on co-existing samples from relatives, participant age range, and location and time period of sample collections. (Many of these data are typically included in a standard ‘methods’ section upon publication.) When this information is combined with public records, re-identification appears to be far more feasible than described by many studies’ consent forms and typically believed by their participants^a^.

The PGP clearly abstains from any assurance to participants of privacy or anonymity, and the ‘open consent’ approach of the project is a significant change from standard human subjects research. The open consent model is based on the perspective that autonomous decision making and valid consent require complete and truthful information (veracity), and that unsustainable promises of anonymity result in invalid consent [5]. With growing concerns regarding identifiability and obvious difficulties in securing data, truthful information about the limitations of privacy protection measures is increasingly seen as a necessary component of informed consent - especially when generating much-needed fully consented public datasets [6].

The non-anonymous approach to creating public genome and health data has led us to a highly participatory project model. As we describe below, our project makes special efforts to educate and test potential participants to ensure that they understand the potential consequences of participation. As a result, participants show a high level of engagement in the project as demonstrated by their high rate of voluntary contributions of data. In addition, we believe public non-anonymous data should imply that participants have access to their research-grade genome data. In keeping with this, we not only return data to participants but also give them access through their project identifier (for example, ‘hu43860C’). Thus, participants have the ongoing ability to follow the research uses of samples and data they have contributed. Although public release of data is difficult to reverse, participants may withdraw at any time and request removal of data and samples from our databases. We also maintain an ongoing relationship with all participants to collect knowledge regarding the consequences of participation. We share here our experiences and recommendations in the hopes of assisting other groups that may be considering similar new research models.

Research at the Harvard PGP discussed herein was approved by the Committee on Human Studies of Harvard Medical School and Harvard School of Dental Medicine (Approval #FWA00007071), supervised by a Data Safety Monitoring Board and conducted in accordance with the principles of the Declaration of Helsinki. Harvard PGP participants named in this manuscript have given informed consent to publicly share their name and their associated participant data outside of the study context of the Personal Genome Project.

## Discussion

### The Personal Genome Project enrollment process

**Figure 1:**
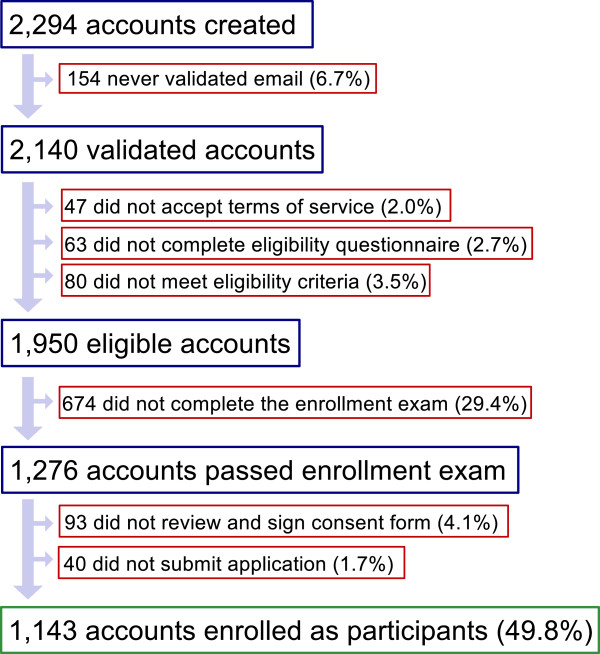
**Status of 2,294 accounts created June 2012 to December 2013.** About half of all accounts created on our site eventually complete the enrollment process to become participants (1,143 users, 50% of all accounts). Of the 1,151 accounts that did not complete the enrollment process, the majority (674 users, or 59% of incomplete enrollments) stopped at the enrollment examination stage.

In society, a wide diversity of preferences exist with respect to levels of privacy, and many individuals choose to participate in the Harvard PGP despite the lack of assurance of privacy and anonymity. Enrollment and participation are very deliberate processes. Prospective participants must first verify their eligibility and, although enrollment is greatly facilitated by an online interface, it nevertheless requires several steps on the part of the participant to demonstrate understanding and consent. Each of these steps accounts for a fraction of potential participants that do not ultimately enroll (Figure 1), and in many cases these are likely individuals who realized that they did not wish to volunteer.

The most notable step in our online enrollment process is our requirement for potential participants to pass an enrollment examination. To ensure the decision to participate is well-informed, we provide a study guide and require individuals to correctly answer all questions on this examination. The examination design is modular (with each module to be repeated until all questions are answered correctly), and both our study guide and consent documents are publicly shared so that other studies may use or adapt them [7]. Our recent data show that the enrollment examination remains the most significant barrier in our online enrollment process: 59% of users who did not complete enrollment in the 2012 to 2013 time period stopped at the enrollment examination stage. About half of the people (49.8%) who created accounts on our site between June 2012 and December 2013 completed the enrollment process (Figure 1). This represents an update on our prior analysis of accounts created until May 2012, which was largely similar, with 41.1% of accounts completing the enrollment process [8]. Among those who passed the examination stage, 90% electronically signed the online consent form and fully enrolled in the project. As of 31 December 2013, 3,181 participants are fully enrolled.

The enrollment examination and the very detailed consent form emphasize the research-only character of the PGP, where participants are not expected to directly benefit. The resulting cohort is therefore enriched for highly motivated individuals interested in contributing to the project, and many of our participant-initiated communications are from participants interested in donating samples as well as genetic and health data they have gathered from external sources (see below).

After enrollment, participants continue to use our website to add data to their public profiles, and to review and publish the data we return to them. Although developing and maintaining the participant-facing infrastructure has been a significant cost, the benefits are apparent. Self-service makes it more practical for participants to exercise their will. Sensitive interactions, such as soliciting feedback during the withdrawal process, are carefully designed and can be consistently executed. The process of encoding the study protocol in the form of software sometimes reveals ambiguities that can be explored and clarified, resulting in better agreement between researchers’ behavior and participants’ expectations. Common interactions like enrollment and sample collection can be largely automated, so the incremental cost of each additional participant is extremely low. With the intention of making our participatory approach more accessible to other research projects, we have released the website software under the GNU General Public License.

### Participant communication

**Figure 2:**
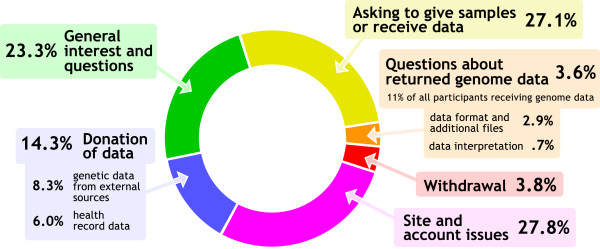
**Participant-initiated communications.** Our website offers participants a ‘Contact Us’ button. From June 2012 to December 2013, we received 579 emails from participants. Few support requests derived from the return of genome data (3.6% of all emails, representing 11% of participants receiving genome data). Most of these were inquiries regarding data formats and additional files for the participants’ own analyses of their data (2.9%), rather than requests for additional interpretation on our part (0.7%).

Participation in the PGP is an ongoing relationship after enrollment. Account and data are managed through our online interface, and participants can use a ‘Contact Us’ button on the website to email us. In the 16 months analyzed here (June 2012 to December 2013; Figure 2), 579 emails were received, which averages about one email per day. Communications were diverse and included general interest and questions (for example, regarding eligibility requirements), interest in donation of data, reports of site bugs and account issues (for example, name changes), and questions about the timeline of sampling and return of data.

As in any study, participants can decide to withdraw at any moment and in the PGP such a decision is not influenced by a patient-physician relationship or opportunities for clinical interventions. Since online enrollment began in 2010, less than 1% of users who have fully enrolled have later withdrawn (26 participants); of the nine participants who shared reasons for withdrawal, five expressed concerns about privacy that developed after enrollment, and four expressed frustration with the timeline and requirements involved for participation. From June 2012 to December 2013, 17 out of 579 emails sent by participants were related to the issue of withdrawal (3.3%, see Figure 2, ‘Withdrawal’). Of the 185 participants who have publicly shared whole genome or exome data, none have withdrawn from the project.

### Participant experiences with the return of genome data

Most projects that create biological data and cell lines do not return data to participants. Samples are typically stripped of identifying data to protect the privacy of participants - although there is increasing recognition that this may not be sufficient to prevent unwanted re-identification, it nevertheless theoretically renders researchers unable to return data to their study participants. Other rationales for not returning data include concerns regarding misuse of data as a clinical tool, and potentially burdensome participant requests for assistance with data interpretation. Modern genotyping and sequencing technologies should cause us to question the coherence of this traditional approach, especially when projects generate public sequence data. Individuals now have ready access to deep genetic data about themselves through direct-to-consumer services, with one million single nucleotide polymorphism datasets available for $100 to $200. The difference between ‘public data’ and ‘access to one’s personal data’ is essentially reduced to the effort a participant must make to identify which public dataset is their own.

Access to and return of data is one of the core components of PGP [9], and the PGP has so far returned whole genome data to 163 individuals. (Our total of 185 includes an additional 22 individuals that have shared genome or exome data obtained elsewhere). We emphasize to participants that our data is research-grade (that is, not for clinical use) and that many types of error are possible, including errors in data, failure to discover or report significant genetic issues, and ambiguous or false positive findings. We also provide access to genome interpretations as produced by the Genomes-Environments-Trait (GET)-Evidence system, which provides a mechanism for continued improvement in genome interpretation and annotation through participant engagement and community review of the scientific literature [8]. Only a small fraction (11%) of participants who received whole genome data have contacted us regarding that data. Of these, only a minority (19%, or 0.8% of total communications) are seeking additional knowledge regarding interpretation, and most (81%, or 3.3% of total communications) are inquiries regarding file formats and access to additional data files, made by participants interested in self-pursuit of additional analysis.

The continued application of our GET-Evidence system has been used to record interpretations of a variety of variants found in participant genomes. These interpretations are publicly shared on the GET-Evidence website [10]. Our overall experience generally continues to be one of ‘false positives’, variants reported to cause phenotypes that the participant does not appear to have. We believe these are generally due to a lack of statistical significance in original literature rather than sequencing errors (notably, sequencing errors are randomly distributed and unlikely to match a previously reported variant).

One false-positive variant that is a useful illustration for the uncertainties in genome interpretation is SCN5A-G615E. This variant was found in a participant who is identified in our public dataset as hu034DB1. Several publications implicate it as a cause of long-QT syndrome. Recommendations released by the American College of Medical Genetics (ACMG) [11] recommend that clinical studies report known pathogenic variants (defined as ‘previously reported and a recognized cause of the disorder’) and expected pathogenic variants (defined as ‘previously unreported and is of the type which is expected to cause the disorder’) in *SCN5A*. How do we determine which variants meet these criteria? A non-skeptical reading of the literature would define variant SCN5A-G615E as a known pathogenic variant. However, we observed that none of these publications demonstrated variant-specific statistically significant enrichment for this variant in cases versus controls. We also confirmed that our participant reported no family history consistent with this disease, and that she pursued clinical evaluation after learning of this variant and was not diagnosed with the disease. Although disease may later manifest in this participant, we have yet to discover a case of unexpected disease in which the causal variant’s pathogenic hypothesis lacked statistical significance. Our experience, in the context of incidental findings, is that the ACMG recommendations provide little guidance when there is no accompanying variant-specific consensus regarding which variants within those genes warrant clinical response.

We also have at least one ‘true positive’ to report: one participant discovered an unanticipated disease after genome sequencing revealed a rare genetic variant. JAK2-V617F, found in a blood sample donated by huA90CE6, is an acquired mutation associated with myeloproliferative disorders^b^. Although this gene is not included in the ACMG recommendations, our evaluation of the literature concluded that a significant fraction of carriers later develop myeloproliferative disorders. Although this participant was not suspected of having any genetic disease, he had a past medical incident involving a blood clot and, upon self-pursued clinical evaluation subsequent to detection of this variant, was discovered to have abnormally high platelets (essential thrombocytosis) and now treats this with low-dose aspirin. The participant, as a journalist, reported this experience in an article series for Bloomberg News [12].

### Participant-contributed data

Our study allows participants to autonomously contribute diverse data to be shared on their public profiles, and many of the emails we receive from participants are inquiries about such contributions (14.3% of emails in the period from June 2012 to December 2013, see Figure 2). To facilitate donation of health records, we have supported import of data from Google Health (now discontinued) and Microsoft Healthvault in Continuity of Care Record format. We parse health conditions from these records for re-display on our site. We would like to share the raw data files themselves, but these files contain sensitive personal data (for example, full names of participants, their health care providers, and email addresses) - even participants open about their account identity may not wish to have all such information publicly shared. In the interest of facilitating future public datasets, we encourage developers of health record management systems to allow individuals to remove their personal identifiers and contact information when exporting records. As of December 2013, 1,235 participants (39% of 3,191 enrolled participants) have contributed health record data through these resources.

Parsing these records gives us a valuable insight into the health and trait data represented in the participant cohort. We recognized, however, that these data can be non-uniform; for example, there are many traits participants may not think to report because they are common or mostly benign. To address this, we created a series of 12 surveys spanning 239 phenotypes (Additional file 1) based on the traits and conditions listed in our health record data. In order to allow for the discovery of unknown associations between variants and hypothesis generation, the range is intentionally broad, ranging from extremely common traits (for example, myopia, dental caries) to moderately rare conditions (for example, porphyria, Marfan syndrome). As of December 2013, 680 participants (21%) have completed all 12 surveys to add trait and disease data to their public profiles. Among the 185 participants who have released whole genome or exome data, 133 (72%) have completed all 12 surveys.

Participant willingness to contribute data extends beyond health data. Many inquiries we receive are from participants interested in donating genetic data acquired elsewhere (8.2% of participant-initiated communications, see Figure 2). As of 31 December 2013, 462 participants have shared through their public profiles genetic data acquired from other sources. This is primarily composed of single nucleotide polymorphism genotyping data, but also includes 22 whole genome and exome datasets.

### Building a participatory research community

Forgoing the assurances of privacy and allowing participants to publicly share identifiable data has shown practical benefits. One important difference we have discovered is that participants are no longer isolated: participants and researchers have been able to meet each other at our yearly GET conference. Participants have also formed participant-managed online groups, including groups on LinkedIn and Facebook and an online forum [13]. The formation of a participant community allows participants to share knowledge, participation experiences, news items of interest, and mutual assistance with the understanding of research data.

Public data inspires important discussions. In January 2013, Gymrek *et al*. used publicly available data from HapMap project samples to demonstrate re-identification methods [4], and later that year another group used our project’s data for similar research [14]. Notably, because these data are public, this research is considered exempt according to exemption 4 of the ‘Common Rule’ of Health and Human Services regulations (45 CFR part 46 subpart A) [15]. No PGP participants withdrew from the project because of these incidents, demonstrating their correct understanding of the public nature of their data with PGP. However, these events highlight a concern for participants in mainstream studies whose data or specimens have been shared publicly and for whom privacy was assured: there is currently no requirement for ethical oversight of re-identification efforts conducted by researchers in the US if they work with publicly available material [16].

Many PGP participants choose to be public about their identity, and some of these have written about the project to share their personal experiences with genome data, as well as broader lessons about genome research and technology. This includes the reporting by John Lauerman mentioned earlier [12], an editorial by Steven Pinker [17], and a book by Misha Angrist [18]. With these writers we can see one of the great potential benefits of participatory research: bridging the divide between researchers and their community to more broadly share scientific understanding.

## Summary

New approaches are needed to create public genome and health data collections. Reflecting this, a growing number of projects interested in creating public data and materials, including the Encyclopedia of DNA Elements project and the National Institute of Standards and Technology Genome In A Bottle consortium, are now working together with the PGP [19]. A tremendous amount of genomics research will be required before clinical utility is established with high confidence on some genetic variants, and studies that include a genomic component may, in aggregate, require the study of very large numbers of individuals before such utility can be established. By promoting non-redundant efforts through public and participant data sharing, participatory research projects like the PGP have the potential to efficiently pave the way to this goal.

We demonstrate that it is feasible for a research study to publicly share combined genomic and health data. When engaged in a participatory manner, participants can be highly motivated to help create these resources: many go beyond volunteering for the uncertainties of public research data, to also volunteer their time and efforts to help create that data. The outcomes for public genome and health data will continue to be explored by our participants and, to date, we have no serious adverse experiences to report. We can also report positive experiences including the ongoing participatory learning experience this project represents for both its participants and researchers.

Recent studies on re-identification of individuals from genomic data reinforce one of the founding premises of the PGP’s open-consent framework. In contrast to research studies in the pre-genomics era, we demonstrate that informed consent regarding the potential for participant re-identification may now be ethically mandated for a wide range of studies in the post-genomics era. We strongly recommend that researchers interested in generating data that is to be publicly shared be clear with their participants about the re-identifiability of that data. This increased transparency may in turn give rise to models that retain relationships with participants, allowing ongoing interaction in the management of the data.

In the process of building our own project, we have also learned some lessons that, although anecdotal, provide insights that may help others facing similar issues. One thing we have learned is that online, automated methods greatly facilitate participant education, enrollment, notification, and provision of access to resulting study data, and allow participants to manage and release data. We have also found that, despite concerns regarding genome interpretation as being costly, its implementation is relatively straightforward in the context of providing participants with access to personal research data. The process is greatly facilitated by automation, and concerns arising from participant access to research data can be addressed through education and careful explanation. Communicating the uncertainties of research and emphasizing potential errors helps to clarify to participants that, while research data may contain incidental findings, these are not clinical data and would need clinical validation to justify any clinical response. Rather than focusing solely on the costs of data interpretation, we recommend research studies planning to return data allocate resources for participant education and communications regarding the process of sample analysis and data creation. Research timelines are much slower, samples analysis is more prone to failure, and the resulting data is less user-friendly than participants would expect from a commercial or medical test.

As we continue to record and report our experiences with public data, return of data and participatory research, we expect the process will continue to improve for both us and other groups.

We believe that openness about the research process in a way that makes it highly interactive helps to communicate the realities of research to participants. Even though participants are not expected to personally benefit from the study, their engagement can significantly benefit the entire research community by providing important feedback that can be used to improve and evolve study designs. It is our hope that the experiences and lessons from the PGP’s open and participatory model will encourage other groups to adopt similar approaches in their research studies.

## Endnotes

^a^From the 1000 Genomes consent form: ‘Because of these measures, it will be very hard for anyone who looks at any of the scientific databases to know which information came from you, or even that any information in the scientific databases came from you’.

^b^This mutation was likely observed because DNA purified from raw blood contains a mixture of tissue sources, including myeloid lineages. We believe it is unlikely this acquired mutation would be observed if genome sequencing is from lymphocyte cell lines or other tissue that does not include myeloid lineages.

## Abbreviations

ACMG: American College of Medical Genetics; GET: Genome-Environment-Trait; PGP: Personal Genome Project.

## Competing interests

MPB, JRB, MFC, TC, PWE, JEL, WV, AWZ and GMC are all members of the Harvard PGP staff. JRB is executive director of PersonalGenomes.org, a 501(c)(3) nonprofit organization supporting the PGP, and MPB is currently receiving compensation for contracting work with PersonalGenomes.org. AWZ, TC and WV are founders of Curoverse, Inc., a company building an open-source platform for managing large biomedical datasets. PWE is founder of TeloMe, a telomere analysis company. GMC has advisory roles in and research sponsorships from several companies involved in genome sequencing technology and personal genomics (http://arep.med.harvard.edu/gmc/tech.html). The authors declare that they have no other competing interests.

## Authors’ contributions

Every author contributed significantly to organizational decisions made by the Harvard PGP. MPB coordinated participant management and return of genome data, authored the current participant trait surveys, and performed the participant communications and user log analyses reported here. JRB coordinates the global PGP network and manages external research programs for the Harvard PGP. MFC manages documentation and incident reports with the project’s Institutional Review Board and Data Safety Monitoring Board. TC developed the informatics infrastructure used for participant enrollment, account/profile management, data release and genome interpretation. PWE coordinated sample collection and management, and genome sequencing. JEL is the PGP ethics consultant and one of the developers of ‘open consent’. WV developed and maintains the informatics infrastructure used for participant enrollment, account/profile management, data release and genome interpretation. AWZ designed the informatics infrastructure used for participant enrollment, account/profile management, data release and genome interpretation. GMC conceived of and leads the project. All authors read and approved the final manuscript.

## Supplementary Material

Additional file 1 Harvard PGP trait and disease surveys. Click here for file
