# Supplementary material for: Harvard Personal Genome Project: lessons from participatory public research
Source: Genome Med. 2014 Feb 28;6(2):10. doi: 10.1186/gm527 (PMC3978420; doi:10.1186/gm527)
Supplement: Additional file 1 — Harvard PGP trait and disease surveys. [file gm527-S1.pdf]

Harvard PGP Trait and Disease Surveys

The following documents represent the surveys made available to Harvard PGP participants in 2012 and 2013. Surveys are administered online through the Google Forms interface, these documents are reproductions of those online forms. Survey responses submitted by participants become publicly available on their public PGP profiles.

These survey questionnaires were authored by Madeleine Price Ball in 2012 and are released as public domain under a CC0 1.0 (or later version) license waiver:
http://creativecommons.org/publicdomain/zero/1.0/

PGP Trait & Disease Survey 2012: Cancers

Answering any or all of these questions is entirely optional. Your answers to this survey will be made publicly available on your participant profile. You are not required to report all conditions when completing a survey.

This survey covers cancers and other abnormal proliferations (ICD-9 codes 140-239, see also: http://en.wikipedia.org/wiki/List_of_ICD-9_codes_140-239:_neoplasms).

Even if you do not have any of the conditions listed here, submitting a response to this form is informative to us -- it indicates that you have seen the survey and are not reporting any of the conditions listed here.

Cancers and other abnormal proliferations

Have you ever been diagnosed with one of the following conditions?

Check any that apply.

[ ] Stomach cancer
[ ] Colon cancer
[ ] Rectal cancer
[ ] Colon polyps
[ ] Pancreatic cancer
[ ] Lung cancer
[ ] Melanoma
[ ] Non-melanoma skin cancer
[ ] Lipoma
[ ] Breast cancer
[ ] Breast fibroadenoma
[ ] Cervical cancer
[ ] Endometrial cancer
[ ] Uterine fibroids
[ ] Ovarian cancer
[ ] Prostate cancer
[ ] Bladder cancer
[ ] Kidney cancer
[ ] Thyroid cancer
[ ] Non-Hodgkin lymphoma
[ ] Leukemia
[ ] Polycythemia vera
[ ] Essential thrombocythemia
[ ] Neurofibromatosis
[ ] Brain cancer

Other condition not listed here?

If you have a condition not listed above that you'd like to report, you may add it here. We can't automatically use data entered in this box, but we'll use these suggestions when we expand our set of surveyed traits in the future.

Publish responses

Thank you for completing this trait survey! By submitting your responses, you are agreeing to make your answers public and connect them to your public PGP profile.

[Submit]

PGP Trait & Disease Survey 2012: Endocrine, metabolic, nutritional, and immune

Answering any or all of these questions is entirely optional. Your answers to this survey will be made publicly available on your participant profile. You are not required to report all conditions when completing a survey.

This survey covers endocrine, metabolic, nutritional, and immune traits and disorders (ICD-9 codes 240-279, see also: http://en.wikipedia.org/wiki/List_of_ICD-9_codes_240-279:_endocrine,_nutritional_and_metabolic_diseases,_and_immunity_disorders). (Note: autoimmune diseases are typically listed in other categories, depending on that disease's physiological effects.)

Even if you do not have any of the conditions listed here, submitting a response to this form is informative to us -- it indicates that you have seen the survey and are not reporting any of the conditions listed here.

Endocrine, metabolic, nutritional, and immune traits and disorders

Have you ever been diagnosed with one of the following conditions?

Check any that apply.

[ ] Thyroid nodule(s)
[ ] Graves' disease
[ ] Hypothyroidism
[ ] Hashimoto's thyroiditis
[ ] Diabetes mellitus, type 1
[ ] Diabetes mellitus, type 2
[ ] Primary hyperparathyroidism
[ ] Growth hormone deficiency
[ ] Polycystic ovary syndrome (PCOS)
[ ] Lactose intolerance
[ ] High cholesterol (hypercholesterolemia)
[ ] High triglycerides (hypertriglyceridemia)
[ ] Alpha 1-antitrypsin deficiency
[ ] Gout
[ ] Hemochromatosis
[ ] Cystic fibrosis
[ ] Porphyria
[ ] Gilbert syndrome

Other condition not listed here?

If you have a condition not listed above that you'd like to report, you may add it here. We can't automatically use data entered in this box, but we'll use these suggestions when we expand our set of surveyed traits in the future.

____

Publish responses

Thank you for completing this trait survey! By submitting your responses, you are agreeing to make your answers public and connect them to your public PGP profile.

[Submit]

PGP Trait & Disease Survey 2012: Blood

Answering any or all of these questions is entirely optional. Your answers to this survey will be made publicly available on your participant profile. You are not required to report all conditions when completing a survey.

This survey covers blood-related traits and disorders (ICD-9 codes 280-289, see also: http://en.wikipedia.org/wiki/List_of_ICD-9_codes_280-289:_diseases_of_the_blood_and_blood-forming_organs).

Even if you do not have any of the conditions listed here, submitting a response to this form is informative to us -- it indicates that you have seen the survey and are not reporting any of the conditions listed here.

Blood traits and disorders

Have you ever been diagnosed with one of the following conditions?

Check any that apply.

- [ ] Iron deficiency anemia
- [ ] Pernicious anemia (a.k.a. "Addison-Biermer anemia")
- [ ] Folate deficiency anemia
- [ ] Hereditary spherocytosis
- [ ] G6PD deficiency
- [ ] Sickle cell trait (carrier)
- [ ] Sickle cell anemia
- [ ] Autoimmune hemolytic anemia
- [ ] Hemophilia
- [ ] Von Willebrand disease
- [ ] Idiopathic thrombocytopenic purpura (ITP)
- [ ] Hereditary thrombophilia (includes Factor V Leiden and Prothrombin G20210A)
- [ ] Other thrombophilia (includes antiphospholipid syndrome)

Other condition not listed here?

If you have a condition not listed above that you'd like to report, you may add it here. We can't automatically use data entered in this box, but we'll use these suggestions when we expand our set of surveyed traits in the future.

____

Publish responses

Thank you for completing this trait survey! By submitting your responses, you are agreeing to make your answers public and connect them to your public PGP profile.

[Submit]

PGP Trait & Disease Survey 2012: Nervous system

Answering any or all of these questions is entirely optional. Your answers to this survey will be made publicly available on your participant profile. You are not required to report all conditions when completing a survey.

This survey covers traits and diseases of the nervous system (ICD-9 codes 320-359, see also: http://en.wikipedia.org/wiki/List_of_ICD-9_codes_320-359:_diseases_of_the_nervous_system).

Even if you do not have any of the conditions listed here, submitting a response to this form is informative to us -- it indicates that you have seen the survey and are not reporting any of the conditions listed here.

Nervous system traits and disorders

Have you ever been diagnosed with one of the following conditions?

Check any that apply.

[ ] Recurrent sleep paralysis
[ ] Parkinson's disease
[ ] Essential tremor
[ ] Huntington's disease
[ ] Restless legs syndrome
[ ] Spinal muscular atrophy
[ ] Amyotrophic lateral sclerosis (ALS)
[ ] Cluster headaches
[ ] Chronic tension headaches (15+ days per month, at least 6 months)
[ ] Multiple sclerosis (MS)
[ ] Cerebral palsy
[ ] Epilepsy
[ ] Migraine with aura
[ ] Migraine without aura
[ ] Narcolepsy
[ ] Arnold-Chiari malformation
[ ] Trigeminal neuralgia
[ ] Bell's palsy
[ ] Carpal tunnel syndrome
[ ] Hereditary motor and sensory neuropathy (includes Charcot-Marie-Tooth disease and HNPP)
[ ] Other peripheral neuropathy
[ ] Muscular dystrophy

Other condition not listed here?

If you have a condition not listed above that you'd like to report, you may add it here. We can't automatically use data entered in this box, but we'll use these suggestions when we expand our set of surveyed traits in the future.

Publish responses

Thank you for completing this trait survey! By submitting your responses, you are agreeing to make your answers public and connect them to your public PGP profile.

[Submit]

PGP Trait & Disease Survey 2012: Vision and hearing

Answering any or all of these questions is entirely optional. Your answers to this survey will be made publicly available on your participant profile. You are not required to report all conditions when completing a survey.

This survey covers traits and diseases of the eye and ear (ICD-9 codes 360-389, see also: http://en.wikipedia.org/wiki/List_of_ICD-9_codes_360-389:_diseases_of_the_sense_organs).

Even if you do not have any of the conditions listed here, submitting a response to this form is informative to us -- it indicates that you have seen the survey and are not reporting any of the conditions listed here.

Vision and hearing traits and disorders

Have you ever been diagnosed with one of the following conditions?

Check any that apply.

- [ ] Retinal detachment
- [ ] Diabetic retinopathy
- [ ] Hypertensive retinopathy
- [ ] Central serous retinopathy
- [ ] Age-related macular degeneration
- [ ] Retinitis pigmentosa
- [ ] Glaucoma
- [ ] Infantile, juvenile, and presenile cataract
- [ ] Age-related cataract
- [ ] Traumatic cataract
- [ ] Hyperopia (Farsightedness)
- [ ] Myopia (Nearsightedness)
- [ ] Astigmatism
- [ ] Presbyopia
- [ ] Color blindness
- [ ] Keratoconus
- [ ] Dry eye syndrome
- [ ] Strabismus
- [ ] Floaters
- [ ] Congenital nystagmus
- [ ] Meniere's disease
- [ ] Otosclerosis
- [ ] Age-related hearing loss
- [ ] Tinnitus
- [ ] Sensorineural hearing loss or congenital deafness

Other condition not listed here?

If you have a condition not listed above that you'd like to report, you may add it here. We can't automatically use data entered in this box, but we'll use these suggestions when we expand our set of surveyed traits in the future.

Publish responses

Thank you for completing this trait survey! By submitting your responses, you are agreeing to make your answers public and connect them to your public PGP profile.

[Submit]

PGP Trait & Disease Survey 2012: Circulatory system

Answering any or all of these questions is entirely optional. Your answers to this survey will be made publicly available on your participant profile. You are not required to report all conditions when completing a survey.

This survey covers traits and diseases of the circulatory system (ICD codes 390-459, see also: http://en.wikipedia.org/wiki/List_of_ICD-9_codes_390-459:_diseases_of_the_circulatory_system).

Even if you do not have any of the conditions listed here, submitting a response to this form is informative to us -- it indicates that you have seen the survey and are not reporting any of the conditions listed here.

Circulatory system traits and disorders

Have you ever been diagnosed with one of the following conditions?

Check any that apply.

[ ] Hypertension
[ ] Myocardial infarction (heart attack)
[ ] Angina
[ ] Pulmonary embolism
[ ] Mitral valve prolapse
[ ] Hypertrophic cardiomyopathy
[ ] Dilated cardiomyopathy
[ ] Restrictive cardiomyopathy
[ ] Other cardiomyopathy (including ARVD)
[ ] Bundle branch block
[ ] Wolff-Parkinson-White (WPW) Syndrome
[ ] Long QT Syndrome
[ ] Heart block
[ ] Atrial fibrillation
[ ] Premature ventricular contractions
[ ] Sick sinus syndrome (includes tachy-brady syndrome)
[ ] Cardiac arrhythmia
[ ] Congestive heart failure
[ ] Stroke
[ ] Aortic aneurysm
[ ] Other aneurysm
[ ] Raynaud's phenomenon
[ ] Kawasaki disease
[ ] Hereditary hemorrhagic telangiectasia (also known as Osler-Weber-Rendu syndrome)
[ ] Deep vein thrombosis (DVT)
[ ] Varicose veins
[ ] Hemorrhoids
[ ] Varicocele

Other condition not listed here?

If you have a condition not listed above that you'd like to report, you may add it here. We can't automatically use data entered in this box, but we'll use these suggestions when we expand our set of surveyed traits in the future.

Publish responses

Thank you for completing this trait survey! By submitting your responses, you are agreeing to make your answers public and connect them to your public PGP profile.

[Submit]

PGP Trait & Disease Survey 2012: Respiratory system

Answering any or all of these questions is entirely optional. Your answers to this survey will be made publicly available on your participant profile. You are not required to report all conditions when completing a survey.

This survey covers traits and diseases of the respiratory system (ICD-9 codes 460-519, see also: http://en.wikipedia.org/wiki/List_of_ICD-9_codes_460-519:_diseases_of_the_respiratory_system).

Even if you do not have any of the conditions listed here, submitting a response to this form is informative to us -- it indicates that you have seen the survey and are not reporting any of the conditions listed here.

Respiratory system traits and disorders

Have you ever been diagnosed with one of the following conditions?

Check any that apply.

[ ] Deviated septum
[ ] Nasal polyps
[ ] Chronic sinusitis
[ ] Chronic tonsillitis
[ ] Allergic rhinitis
[ ] Chronic bronchitis
[ ] Emphysema
[ ] Asthma
[ ] Chronic Obstructive Pulmonary Disease (COPD)

Other condition not listed here?

If you have a condition not listed above that you'd like to report, you may add it here. We can't automatically use data entered in this box, but we'll use these suggestions when we expand our set of surveyed traits in the future.

Publish responses

Thank you for completing this trait survey! By submitting your responses, you are agreeing to make your answers public and connect them to your public PGP profile.

[Submit]

PGP Trait & Disease Survey 2012: Digestive system

Answering any or all of these questions is entirely optional. Your answers to this survey will be made publicly available on your participant profile. You are not required to report all conditions when completing a survey.

This survey covers traits and diseases of the digestive system (ICD-9 codes 520-579, see also: http://en.wikipedia.org/wiki/List_of_ICD-9_codes_520-579:_diseases_of_the_digestive_system).

Even if you do not have any of the conditions listed here, submitting a response to this form is informative to us -- it indicates that you have seen the survey and are not reporting any of the conditions listed here.

Digestive system traits and disorders

Have you ever been diagnosed with one of the following conditions?

Check any that apply.

- [ ] Impacted tooth
- [ ] Dental cavities
- [ ] Gingivitis
- [ ] Temporomandibular joint (TMJ) disorder
- [ ] Canker sores (oral ulcers)
- [ ] Geographic tongue
- [ ] Fissured tongue
- [ ] Gastroesophageal reflux disease (GERD)
- [ ] Barrett's esophagus
- [ ] Peptic ulcer (stomach or duodenum)
- [ ] Appendicitis
- [ ] Inguinal hernia
- [ ] Hiatal hernia
- [ ] Crohn's disease
- [ ] Ulcerative colitis
- [ ] Diverticulosis
- [ ] Irritable bowel syndrome (IBS)
- [ ] Rectal prolapse
- [ ] Acute liver failure
- [ ] Chronic liver disease and cirrhosis
- [ ] Nonalcoholic fatty liver disease (NAFLD)
- [ ] Gallstones
- [ ] Celiac disease

Other condition not listed here?

If you have a condition not listed above that you'd like to report, you may add it here. We can't automatically use data entered in this box, but we'll use these suggestions when we expand our set of surveyed traits in the future.

Publish responses

Thank you for completing this trait survey! By submitting your responses, you are agreeing to make your answers public and connect them to your public PGP profile.

[Submit]

PGP Trait & Disease Survey 2012: Genitourinary systems

Answering any or all of these questions is entirely optional. Your answers to this survey will be made publicly available on your participant profile. You are not required to report all conditions when completing a survey.

This survey covers traits and diseases of the genitourinary systems (ICD-9 codes 580-629, see also: http://en.wikipedia.org/wiki/List_of_ICD-9_codes_580-629:_diseases_of_the_genitourinary_system).

Even if you do not have any of the conditions listed here, submitting a response to this form is informative to us -- it indicates that you have seen the survey and are not reporting any of the conditions listed here.

Genitourinary systems traits and disorders

Have you ever been diagnosed with one of the following conditions?

Check any that apply.

[ ] Kidney stones
[ ] Acute kidney failure
[ ] Chronic kidney failure
[ ] Urinary tract infection (UTI)
[ ] Urethral diverticulum
[ ] Benign prostatic hypertrophy (BPH)
[ ] Male infertility
[ ] Peyronie's disease
[ ] Spermatocele
[ ] Fibrocystic breast disease
[ ] Bartholin's cyst
[ ] Endometriosis
[ ] Uterine prolapse
[ ] Ovarian cysts
[ ] Female infertility

Other condition not listed here?

If you have a condition not listed above that you'd like to report, you may add it here. We can't automatically use data entered in this box, but we'll use these suggestions when we expand our set of surveyed traits in the future.

____

Publish responses

Thank you for completing this trait survey! By submitting your responses, you are agreeing to make your answers public and connect them to your public PGP profile.

[Submit]

PGP Trait & Disease Survey 2012: Skin and subcutaneous tissue

Answering any or all of these questions is entirely optional. Your answers to this survey will be made publicly available on your participant profile. You are not required to report all conditions when completing a survey.

This survey covers traits and diseases of the skin and subcutaneous tissue (ICD-9 codes 680-709, see also: http://en.wikipedia.org/wiki/List_of_ICD-9_codes_680-709:_diseases_of_the_skin_and_subcutaneous_tissue).

Even if you do not have any of the conditions listed here, submitting a response to this form is informative to us -- it indicates that you have seen the survey and are not reporting any of the conditions listed here.

Skin and subcutaneous tissue traits and disorders

Have you ever been diagnosed with one of the following conditions?

Check any that apply.

[ ] Pilonidal cyst
[ ] Dandruff
[ ] Eczema
[ ] Allergic contact dermatitis
[ ] Rosacea
[ ] Psoriasis
[ ] Lichen planus
[ ] Keloids
[ ] Skin tags
[ ] Hair loss (includes female and male pattern baldness)
[ ] Alopecia areata
[ ] Hyperhidrosis (excessive sweating)
[ ] Hidradenitis suppurativa
[ ] Acne
[ ] Dermatographia
[ ] Cafe au lait spots

Other condition not listed here?

If you have a condition not listed above that you'd like to report, you may add it here. We can't automatically use data entered in this box, but we'll use these suggestions when we expand our set of surveyed traits in the future.

Publish responses

Thank you for completing this trait survey! By submitting your responses, you are agreeing to make your answers public and connect them to your public PGP profile.

[Submit]

PGP Trait & Disease Survey 2012: Musculoskeletal system and connective tissue

Answering any or all of these questions is entirely optional. Your answers to this survey will be made publicly available on your participant profile. You are not required to report all conditions when completing a survey.

This survey covers traits and diseases of the musculoskeletal system and connective tissue (ICD-9 codes 710-739, see also: http://en.wikipedia.org/wiki/List_of_ICD-9_codes_710-739:_diseases_of_the_musculoskeletal_system_and_connective_tissue).

Even if you do not have any of the conditions listed here, submitting a response to this form is informative to us -- it indicates that you have seen the survey and are not reporting any of the conditions listed here.

Musculoskeletal system and connective tissue traits and disorders

Have you ever been diagnosed with one of the following conditions?

Check any that apply.

- [ ] Lupus
- [ ] Sjogren's syndrome (Sicca syndrome)
- [ ] Rheumatoid arthritis
- [ ] Osteoarthritis
- [ ] Chondromalacia patella (CMP)
- [ ] Spinal stenosis
- [ ] Sciatica
- [ ] Frozen shoulder
- [ ] Rotator cuff tear
- [ ] Tennis elbow
- [ ] Achilles tendonitis
- [ ] Bone spurs
- [ ] Trigger finger
- [ ] Bunions
- [ ] Dupuytren's contracture
- [ ] Plantar fasciitis
- [ ] Fibromyalgia
- [ ] Scheuermann's kyphosis
- [ ] Osgood-Schlatter disease
- [ ] Osteoporosis
- [ ] Flatfeet
- [ ] Postural kyphosis
- [ ] Scoliosis

Other condition not listed here?

If you have a condition not listed above that you'd like to report, you may add it here. We can't automatically use data entered in this box, but we'll use these suggestions when we expand our set of surveyed traits in the future.

Publish responses

Thank you for completing this trait survey! By submitting your responses, you are agreeing to make your answers public and connect them to your public PGP profile.

[Submit]

PGP Trait & Disease Survey 2012: Congenital traits and anomalies

Answering any or all of these questions is entirely optional. Your answers to this survey will be made publicly available on your participant profile. You are not required to report all conditions when completing a survey.

This survey covers congenital traits and anomalies (ICD-9 codes 740-759, see also: http://en.wikipedia.org/wiki/List_of_ICD-9_codes_740-759:_Congenital_anomalies).

Even if you do not have any of the conditions listed here, submitting a response to this form is informative to us -- it indicates that you have seen the survey and are not reporting any of the conditions listed here.

Congenital traits and anomalies

Have you ever been diagnosed with one of the following conditions?

Check any that apply.

[ ] Spina bifida
[ ] Congenital ocular coloboma
[ ] Congenital heart defect
[ ] Cleft palate
[ ] Cleft uvula
[ ] Cleft lip
[ ] Tongue tie (ankyloglossia)
[ ] Bifid tongue (cleft tongue)
[ ] Infantile pyloric stenosis
[ ] Hirschsprung's disease
[ ] Hypospadias
[ ] Renal agenesis (missing kidney)
[ ] Polycystic kidney disease
[ ] Congenital hydronephrosis
[ ] Developmental dysplasia of the hip
[ ] Congenital clubfoot (equinovarus)
[ ] Polydactyly
[ ] Syndactyly (webbing of digits)
[ ] Ehlers-Danlos syndrome
[ ] Congenital ichthyosis
[ ] Single transverse palmar crease (simian crease)
[ ] Marfan syndrome

Other condition not listed here?

If you have a condition not listed above that you'd like to report, you may add it here. We can't automatically use data entered in this box, but we'll use these suggestions when we expand our set of surveyed traits in the future.

Publish responses

Thank you for completing this trait survey! By submitting your responses, you are agreeing to make your answers public and connect them to your public PGP profile.

[Submit]
